# The short term influence of right ventricular pacing burden on echocardiographic and spiroergometric parameters in patients with preserved left ventricular ejection fraction

**DOI:** 10.1186/s12872-021-02429-0

**Published:** 2022-01-31

**Authors:** Akram Youssef, Christian Pfluecke, Maciej Dawid, Karim Ibrahim, Michael Günther, Steffen Kolschmann, Utz Richter, Alexander Francke, Carsten Wunderlich, Marian Christoph

**Affiliations:** 1 Technische Universität Dresden, (Campus Chemnitz), Klinikum Chemnitz, Flemmingstrasse 2, 09116 Chemnitz, Germany; 2 Technische Universität Dresden, University of Dresden, Fetscherstrasse 76, 01307 Dresden, Germany; 3 HELIOS Hospital Pirna: HELIOS Klinikum Pirna, Struppener Strasse 13, 01796 Pirna, Germany

**Keywords:** Ventricular pacing, Pacemaker, Echocardiography, Spiroergometry, Clinical outcome

## Abstract

**Background:**

The incidence of worsened clinical outcome due to high right ventricular (RV) pacing burden in patients with preserved left ventricular function remains controversial.

**Objective:**

To investigate the impact of RV pacing on several echocardiographic and spiroergometric parameters.

**Methods:**

In 60 pacemaker patients with preserved left ventricular ejection fraction (LVEF) serial echocardiographies and spiroergometries were performed over a time course of 12 months. Additionally, in 48 patients retrospective echocardiographic analyses of the LV- and RV function were carried out up to 24 months after pacemaker implantation.

**Results:**

The patients were divided into two groups: The high RV pacing burden group (hRVP: ≥ 40%) and the low RV pacing group (lRVP < 40%) according to the definitions in previous randomized MOST and DAVID trials. After a period of 12-month pacemaker therapy no changes to left ventricular end diastolic diameter (LVEDD), left ventricular end systolic diameter (LVESD), LVEF, E/A-ratio; E/E′-ratio and tricuspid annular plane systolic excursion (TAPSE) could be revealed, independently of the RV pacing burden. Additionally, after 24-month long term follow-up there were no differences in LVEF and TAPSE in both groups. Accordingly, no relevant changes of peak exercise capacity, ventilatory anaerobic threshold or maximal oxygen consumption could be demonstrated independently of the RV pacing.

**Conclusions:**

In pacemaker patients with preserved LVEF the burden of RV pacing has no adverse influence on several echocardiographic and spiroergometric surrogate parameters of pacemaker-induced cardiomyopathy after a follow-up of 12 to 24 month. Despite this, screening for pacemaker induced cardiomyopathy should be performed especially in the presence of new heart failure symptoms.

## Background

The incidence of pacemaker induced cardiomyopathy as the cause of worsened clinical outcome due to high right ventricular (RV) pacing burden remains unclear, especially in patients with preserved left ventricular function [1]. A previous large single centre registry of left ventricular ejection fraction (LVEF) as an echocardiographic parameter for pacemaker-induced cardiomyopathy and death from any cause as clinical outcome parameter depending on RV pacing burden in patients with preserved or mildly reduced left ventricular (LV) function could not reveal a predictive value of RV pacing burden [2]. In addition, a large registry of the Mayo Clinic could not observe any statistically significant change in mean LVEF in long-term follow-up of patients with initially preserved LVEF who underwent atrioventricular junction ablation and RV pacing [3]. However, in another large registry by Bansal et al. the incidence of pacemaker-induced cardiomyopathy was found to be 13.8% over a mean follow-up of 14.5 months [4]. The clinical relevance of the proposed pacemaker-induced cardiomyopathy was predominantly investigated in patients with reduced LVEF. These studies suggest an adverse clinical effect of RV pacing. With regard to clinical endpoints, the DAVID trial and the MOST trial revealed increased rates of death or hospitalization for heart failure and increased incidence of atrial fibrillation in patients with a cumulative RV pacing > 40% [5–7]. In this context, biventricular pacing has been controversially postulated as a preventive method. However, no benefits of such an approach could be found in the randomized prospective PREVENT-HF study which compared biventricular with RV apical pacing with regard to several echocardiographic parameters [8]. On the other hand, the advantages of biventricular pacing for LVEF could be demonstrated in the PACE trial [9]. Further, HIS bundle pacing is currently being investigated for the prevention of pacemaker-induced cardiomyopathy in various studies with different patient populations.

In the present clinical registry we evaluated the influence of RV pacing burden on several echocardiographic parameters. In addition, serial spiroergometric examinations were performed to investigate the clinically functional outcome of the pacemaker patients. The aim was to complement the existing data in order better to evaluate the need for screening for pacemaker-induced cardiomyopathy and potentially verify the indication for biventricular pacing and HIS bundle pacing in patients with preserved LV function.

## Methods

### Study design

The current study was a single-centre registry, divided into a retrospective and a prospective part, performed in compliance with the guidelines for good clinical practice and the Declaration of Helsinki. The study was approved by the institutional ethical review board. All data were collected, managed and analysed at the Heart Centre, University of Dresden (ethics votum University of Dresden: EK 28409202).

The *primary endpoints* of this study were the left ventricular end diastolic and end systolic diameter (LVEDD, LVESD), the systolic left ventricular function (LVEF), the diastolic left ventricular function (E/A-ratio; E/E′-ratio) and the systolic right ventricular function (tricuspid annular plane systolic excursion, TAPSE) in dependence of the RV apical pacing burden.

The *secondary clinical endpoints were* the changes in maximal oxygen consumption (VO_2_max), maximal workload and ventilatory anaerobic threshold (VAT) in dependence of the RV apical pacing burden.

### Study population and protocol

Eligible subjects were consecutive males or females > 18 years of age with an indication for permanent pacemaker therapy according to the European guidelines [10]. The main indications for pacemaker implantation were symptomatic sinus node dysfunction, paroxysmal or permanent second-degree type II or third-degree AV block including symptomatic bradyarrhythmia during persistent atrial fibrillation or unexplained syncope with bundle branch block or with positive electrophysiological study (HV-interval > 70 ms).

Consecutive patients who received a pacemaker from May 2010 to December 2011 and received all clinical routine follow-up visits in the study centre were included in the retrospective analysis.

The study centre is the University Hospital Dresden Heart Centre. At the time of the study, approximately 250 pacemakers per year were implanted by the co-authors MG and SK. Of these 250 patients, approximately 50 patients per year were completely followed up as part of the routine clinical practice at the university outpatient clinic. From these patients, those who had normal baseline LVEF and no other cardiac cause of worsening cardiac function besides pacemaker therapy were selected consecutively for the registry.

After the analysis of the 24-month follow-up data, the expansion to include the prospective part of the registry was done. Prospective inclusion in the registry was conducted from September 2014 to June 2015. The inclusion criteria were as follows: 1. Normal left ventricular ejection fraction > 55%, 2. Normal to mildly dilated left ventricular end diastolic diameter < 57 mm, 3. Normal right ventricular function TAPSE > 17 mm, 4. Exclusion of other cardiac causes of worsened LV function and spiroergometric parameters. If a patient fulfilled all inclusion criteria, primarily a retrospective evaluation of clinical routine echocardiographic parameters (LVEF and TAPSE) was conducted at the time of pacemaker implantation and after 24 months in 48 consecutive pacemaker patients. As there were no changes in this routine echocardiographic parameters in the retrospective analysis over 24 months, a prospective part was added to the registry in which the 6 and 12 month follow-up visits were expanded to include additional echocardiographic parameters (LVEDD, LVESD, LVEF, E/A-ratio; E/E′-ratio and TAPSE) and spiroergometry (VO_2_max, peak exercise capacity, VAT).

The measurements of the echocardiographic and spiroergometric data during the follow-up visits were performed by independent staff who did not know that the data were registered. Additionally, the staff was not informed about the RV pacing burden of the patients.

### Pacemaker implantation, programming and follow-up

The type of pacemaker to be implanted was selected by the responsible physician according to bradyarrhythmia type and the current guidelines [10]. All RV pacing leads were exclusively positioned in the RV apex. The correct positioning of implanted leads in the right ventricular septal apex was confirmed by fluoroscopy in RAO and LAO view. The pacemakers were programmed immediately after implantation according to the pacemaker indication and guidelines. The first pacemaker follow-up was performed 4 weeks postoperatively. Further monitoring as well as recording of the RV apical pacing burden took place after 6, 12 and 24 months.

### Transthoracic echocardiography

In all subjects an echocardiogram was performed to assess LVEDD, LVESD, LVEF, E/A-ratio; E/E′-ratio and TAPSE at the time of enrollment, which was repeated at 6 and 12 months after pacemaker implantation. In the retrospective study group, LVEF and TAPSE were analyzed at the time of pacemaker implantation and 24 month afterwards. For the echocardiographic examinations, the iE33 xMatrix-DS ultrasound device from Philips (Koninklijke Philips N. V., Amsterdam, The Netherlands) with a 2.5 MHz transducer was used. All acquired echocardiograms were reviewed by a core laboratory at the Heart Center Dresden. The LVEDD and LVESD was measured M-mode guided in the parasternal long-axis view. The LVEF was assessed using biplane Simpson’s rule with manual tracing of digital images [11]. TAPSE was determined with M-mode guiding in the apical 4-chambers view. Thereby, the maximal systolic excursion of the RV free wall at the junction with the tricuspid valve was measured [12]. The evaluation of LV diastolic function was determined, measuring the mitral inflow and DTI of the mitral lateral annulus. The E and A peaks as well as the *e*′ and *a*′ peaks were measured, followed by the calculation of the E/A-ratio and E/E′-ratio [13].

### Spiroergometry

In all subjects a spiroergometry was performed to assess maximal oxygen consumption (VO_2_max), peak exercise capacity and ventilatory anaerobic threshold (VAT) at the time of enrollment, which was repeated at 6 and 12 months after pacemaker implantation.

After a 5-min rest period, the patients were subjected to an examination on a bicycle ergometer. During exercise a 12-lead ECG was recorded and the heart rate was continuously calculated based on R–R interval. The arterial blood pressure was measured at rest and at least once at a given resistance level. A ramp protocol was employed with continuously increasing resistance of 25 W every 2 min starting at 0 W. Subjects were exercised to fatigue or until one of the following termination criteria was met: chest pain or dizziness, potentially dangerous arrhythmias or ST-segment deviations, marked systolic hypotension or hypertension [14]. During the test, the patient breathed through a tight-fitting mask. The inhaled and the exhaled air volume and flow were measured by a connected spirometer. Additionally, the oxygen consumption (VO_2_) and carbon dioxide production (VCO_2_) were quantified. Maximal oxygen consumption (VO_2_max) was defined as the highest VO_2_ obtained during the entire test and expressed in ml/min/kg [15]. The ventilator anaerobic threshold VAT was determined as the first non-linear increase in the ventilatory equivalent for oxygen without a simultaneous increase of the ventilatory equivalent for CO_2_ [16].

### Statistical analysis

Data were tested for normal distribution. Results of continuous variables are expressed as means ± standard error of the mean. Statistical analyses were done using the 2 tailed, unpaired Student’s t-test. Level of significance was set to *p* < 0.05. Categorical variables are presented as total number with comparison using chi-square statistics and Fisher exact test. If more than 2 groups were analyzed, a one-way ANOVA test was performed. Post hoc analyses were applied using Bonferroni method. Significance level was set to *p* < 0.05.

## Results

### Study population and procedural data

**Fig. 1:**
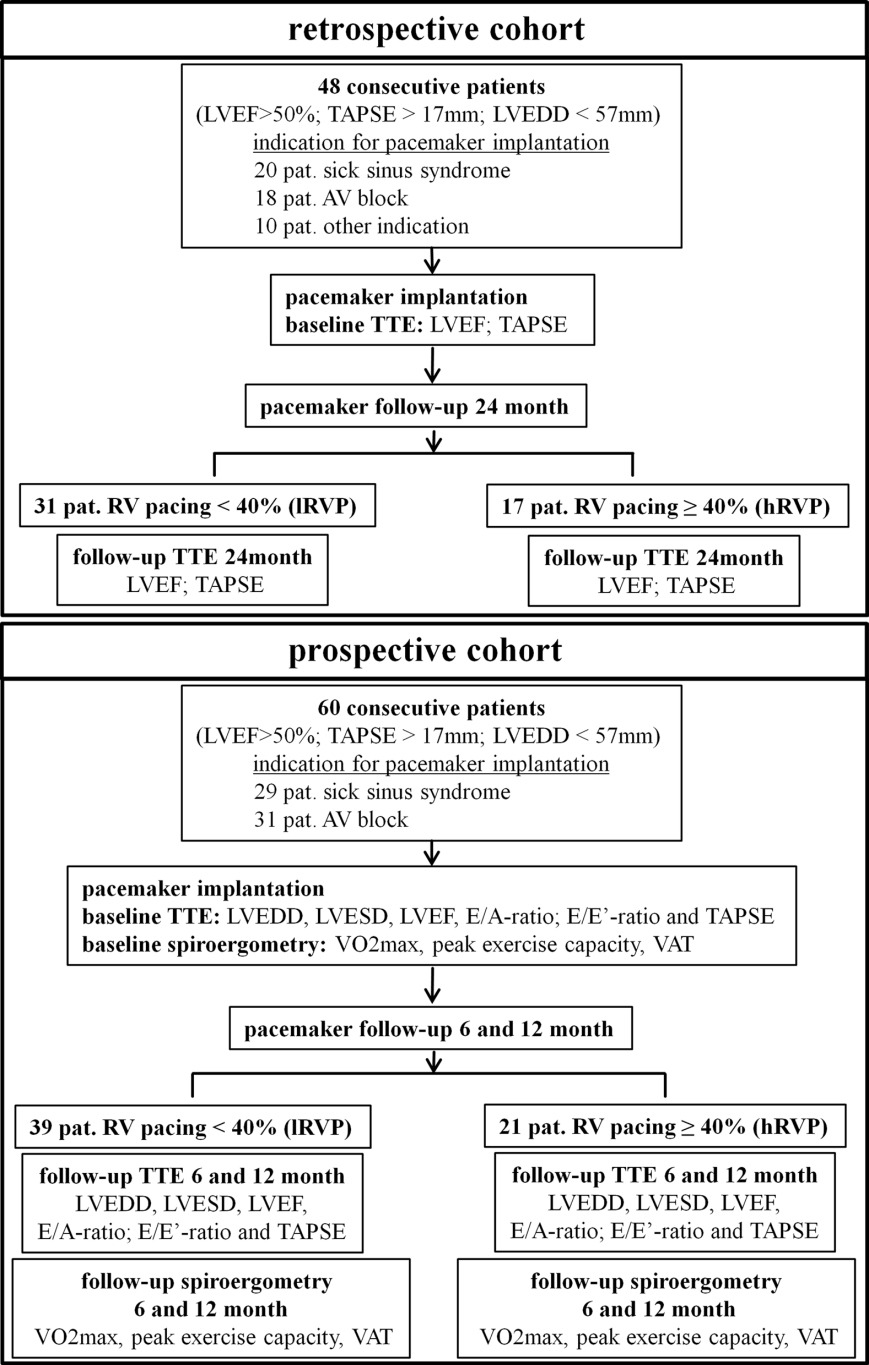
Patient flow chart for the retrospective and the prospective cohort. RV pacing: right ventricular pacing; lRVP: RV pacing < 40%; hRVP: RV pacing ≥ 40%; TTE: transthoracic echocardiogram; LVEDD: left ventricular end diastolic diameter in mm; LVESD: left ventricular end systolic diameter in mm; LVEF: left ventricular ejection fraction in %; TAPSE: tricuspid annular plane systolic excursion; diastolic function E/A ratio; diastolic function E/E′ ratio; peak exercise capacity in watt; VO_2_max: maximal oxygen consumption in ml/min/kg; VAT: ventilator anaerobic threshold in watt

Primary 48 patients were included in the retrospective analysis for the 24 month long time follow-up. In addition, 60 consecutive patients were included in the current prospective 12-month short time analysis for a more detailed echocardiographic and spiroergometric evaluation (Fig. 1).

The patients were divided into two groups. The high RV pacing burden group (hRVP) was defined as subjects with a RV pacing ≥ 40% according to the definitions in previous randomized trials [7]. In contrast, the low RV pacing burden group (lRVP) was defined as RV pacing < 40%. The two treatment groups were well balanced in regards to demographics and clinical baseline characteristics for the retrospective and prospective registry. These are shown in Table 1. In the retrospective cohort the patients in the hRVP group were significantly older compared to the lRVP subjects (58 years vs. 72 years; *p* < 0.05). Additionally, in the retrospective cohort notably more dual-chamber pacemakers were implanted in the lRVP patients. There were no relevant differences in gender, co-morbidities and concomitant medications in both groups.

**Table 1:** Baseline characteristics

	RV-pacing	lRVP	hRVP	*p* value
(Prospective)	Total population	39	21	
	Age, year	71 (11.4)	72 (12.4)	0.6
	Sex, male N (%)	21 (53.8)	14 (66.7)	0.4
	Comorbidities			
	Hypertension N (%)	34 (87.2)	18 (85.7)	1.0
	Diabetes mellitus N (%)	22 (56.4)	14 (66.7)	0.58
	CAD N (%)	24 (63.2)	15 (71.4)	0.57
	A Fib N (%)	5 (12.8)	7 (33.3)	0.09
	Medications			
	Beta blocker N (%)	34 (87.2)	18 (85.7)	1.0
	ACE inhibitor N (%)	34 (87.2)	34 (87.2)	1.0
	Pacemaker indication			
	SSS N (%)	29 (74.4)	0(0)	**< 0.05**
	AV-block N (%)	10 (25.6)	21(100)	
	Type of pacemaker			
	Single-chamber N (%)	2 (5.1%)	5 (23. 8%)	0.25
	Dual-chamber N (%)	37 (94.9)	16 (76. 2%)	
(Retrospective)	Total population	31	17	
	Age, year	58 (13.5)	72 (6.1)	**< 0.05**
	Sex, male N (%)	19 (61.3)	8 (47.1)	0.37
	Comorbidities			
	Hypertension N (%)	26 (83.9)	16 (94.1)	0.40
	Diabetes mellitus N (%)	10 (32.3)	5 (29.4)	1.0
	CAD N (%)	19 (61.3)	14 (82.4)	0.196
	A Fib N (%)	1 (3.2)	7 (41.2)	**< 0.05**
	Medications			
	Beta blocker N (%)	26 (83.9)	17 (100)	0.14
	ACE inhibitor N (%)	26 (83.9)	16 (94.1)	0.40
	Pacemaker indication			
	SSS N (%)	19 (61.3)	1 (5.9)	**< 0.05**
	AV-block N (%)	3 (9.7)	15 (88.2)	
	Other N (%)	9 (29)	1 (5.9)	
	Type of pacemaker			
	Single-chamber N (%)	0 (0)	5 (29.4)	**< 0.05**
	Dual-chamber N (%)	31 (100)	12 (70.6)	

The statistical significance level was set to p < 0.05. Therefore all p- values that reach this level were marked in bold.

RV-pacing: right ventricular pacing; lRVP: RV pacing < 40%; hRVP: RV pacing ≥ 40%

CAD: coronary artery disease; A Fib: atrial fibrillation; SSS: sick sinus syndrome; AV-Block: atrioventricular block; ACE-Inhibitor: angiotensin-converting enzyme; Other pacemaker indications: bundle branch block and syncope or syncope with positive electrophysiological study (HV-interval > 70 ms)

The RV pacing burden was registered at the following routine pacemaker checkups: 4 weeks after pacemaker implantation as well as 6, 12 and 24 months after implantation. No crossover patients from the hRVP to the lRVP group during the follow-up were identified. In the retrospective analysis the average RV pacing burden was 12.3% in the lRVP group versus 75.5% in the hRVP group. In the prospective part of the registry the RV pacing burden was 9.6% in the lRVP patients vs. 78.9% in the hRVP group.

Further results are evaluated below separately for the retrospective and prospective parts of the registry.

#### Retrospective 24-month follow-up

In the retrospective cohort 88.2% of patients in the hRVP group received a pacemaker due to an AV-block, 5.9% pacemakers were implanted because of a symptomatic sick sinus syndrome and 5.9% of patients suffered from syncope with bundle branch block. In contrast, only 9.7% of patients in the lRVP group suffered from an AV-block, 61.3% suffered from sick sinus syndrome and 29% from syncope with bundle branch block (Table 1).

#### Change of echocardiographic parameters depending on the RV pacing burden (primary endpoint)

**Fig. 2:**
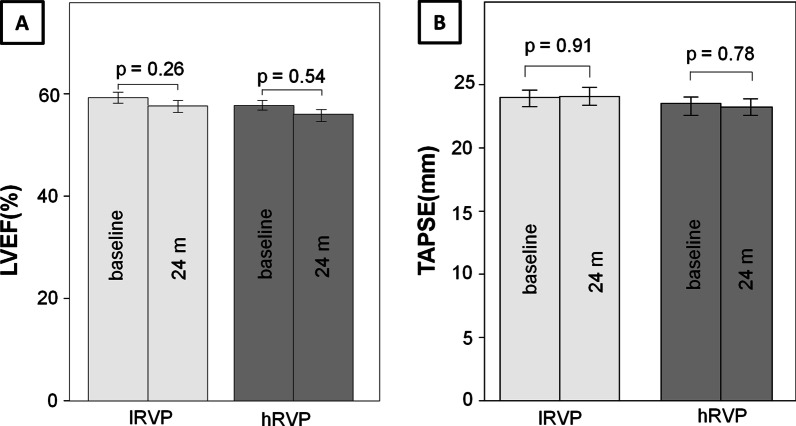
Time course of echocardiographic parameters during RV pacing (retrospective analyses). RV pacing: right ventricular pacing; lRVP: RV pacing < 40%; hRVP: RV pacing ≥ 40%; 24 m: after 24 months; **A** LVEF: left ventricular ejection fraction in %; **B** TAPSE: tricuspid annular plane systolic excursion

The changes in the echocardiographic parameters over the course of 24 months depending on the RV pacing burden are shown in Fig. 2 for the retrospective cohort. In short, none of the measured echocardiographic parameters differed significantly in the course of 24 months, independently of the burden of RV pacing (*LVEF:* lRVP: baseline: 58.2 ± 0.82%, 24 months: 56.7 ± 0.99%; hRVP: baseline: 56.8 ± 1.2%, 24 months: 55.4 ± 2.0%; *TAPSE:* lRVP: baseline: 24.0 ± 0.48 mm, 24 months: 24.1 ± 0.34 mm hRVP: baseline: 23.5 ± 0.86 mm, 24 months: 23.2 ± 0.66 mm).

#### Prospective 6- and 12-month follow-up

In the prospective cohort all patients suffered from AV-block in the hRVP group. In the lRVP group this was the case in 25.6% of patients whereas 74.4% had sick sinus syndrome (Table 1).

#### Change in echocardiographic parameters depending on the RV pacing burden (primary endpoint)

**Fig. 3:**
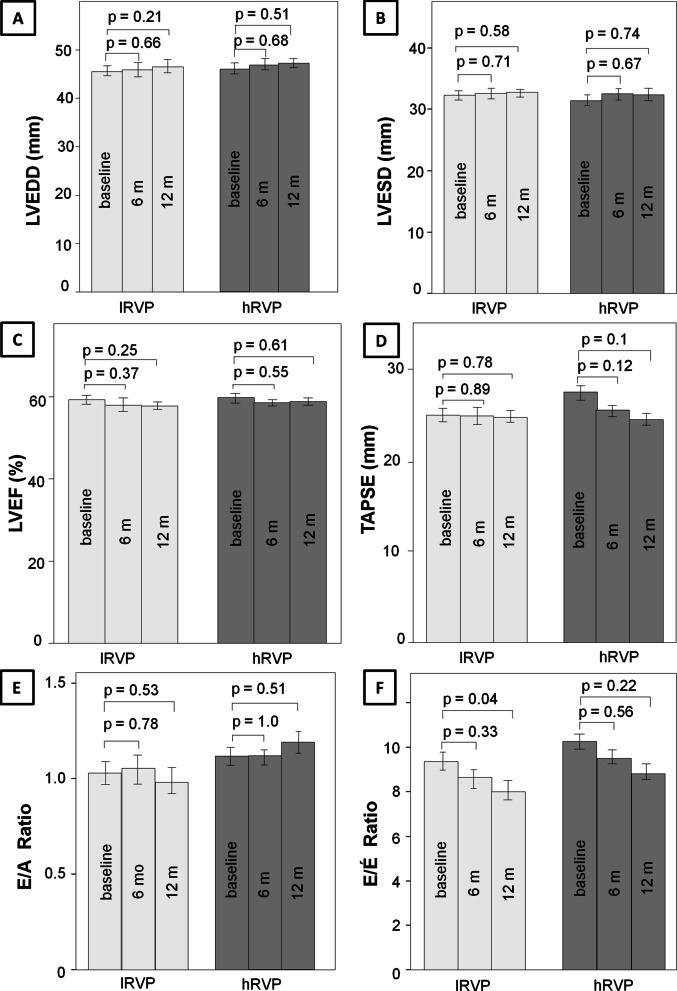
Time course of echocardiographic parameters during RV pacing (prospective analyses). RV pacing: right ventricular pacing; lRVP: RV pacing < 40%; hRVP: RV pacing ≥ 40%; 6 m: after 6 months; 12 m: after 12 months; **A** LVEDD left ventricular end diastolic diameter in mm; **B** LVESD left ventricular end systolic diameter in mm; **C** LVEF: left ventricular ejection fraction in %; **D** TAPSE: tricuspid annular plane systolic excursion; **E** diastolic function E/A ratio; **F** diastolic function E/E′ ratio

The changes in more detailed echocardiographic parameters over the course of one year depending on the RV pacing burden is shown in Fig. 3 for the prospective cohort. These echocardiographic parameters also did not change significantly in the time course of 6 and 12 months, independently of the burden of RV pacing (*LVEDD:* lRVP: baseline: 45.4 ± 0.64 mm, 6 months: 45.5 ± 0.58 mm, 12 months: 46.4 ± 0.51 mm, hRVP: baseline: 45.6 ± 1.4 mm, 6 months: 46.5 ± 1.1 mm, 12 months: 47.2 ± 1.0 mm; *LVESD*: lRVP: baseline: 32.2 ± 0.74 mm, 6 months: 32.6 ± 0.62 mm, 12 months: 32.8 ± 0.61 mm, hRVP: baseline: 31.7 ± 1.5 mm, 6 months: 32.6 ± 1.2 mm, 12 months: 32.4 ± 1.1 mm; *LVEF:* lRVP: baseline: 59.2 ± 1.0%, 6 months: 57.9 ± 0.93%, 12 months: 57.6 ± 0.77; hRVP: baseline: 59.7 ± 1.6%, 6 months: 58.5 ± 1.1%, 12 months: 58.8 ± 0.9%; *TAPSE:* lRVP: baseline: 23.7 ± 0.71 mm, 6 months: 23.5 ± 0.76 mm, 12 months: 23.4 ± 0.57 mm; hRVP: baseline: 26.0 ± 0.85 mm, 6 months: 24.2 ± 0.76 mm, 12 months: 23.3 ± 0.58 mm; *E/A-ratio:* lRVP: baseline: 1.01 ± 0.05, 6 months: 1.05 ± 0.05, 12 months: 0.98 ± 0.04, hRVP: baseline: 1.1 ± 0.07, 6 months: 1.1 ± 0.06, 12 months: 1.19 ± 0.08; *E/E′-ratio:* lRVP: baseline: 9.3 ± 0.5, 6 months: 8.6 ± 0.44, 12 months: 8.0 ± 0.31, hRVP: baseline: 10.2 ± 1.0, 6 months: 9.5 ± 0.77, 12 months: 8.8 ± 0.59).

#### Change in spiroergometric parameters depending on the RV pacing burden (secondary endpoint)

**Fig. 4:**
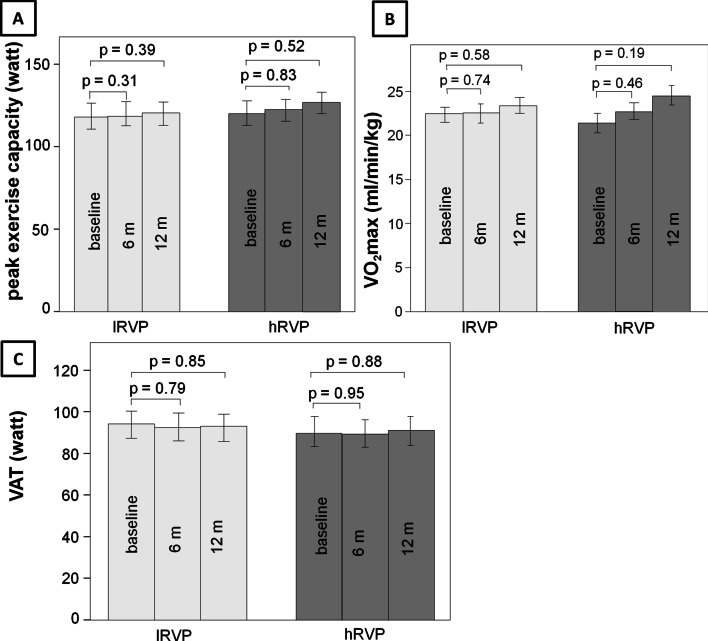
Time course of spiroergometric parameters during RV pacing (prospective analyses). RV pacing: right ventricular pacing; lRVP: RV pacing < 40%; hRVP: RV pacing ≥ 40%; 6 m: after 6 months; 12 m: after 12 months; **A** peak exercise capacity in watt; **B** VO_2_max: maximal oxygen consumption in ml/min/kg; **C** VAT: ventilator anaerobic threshold in watt

Figure 4 shows the changes in spiroergometric parameters over the course of one year depending on the RV pacing burden in the prospective group. Analogous to the echocardiographic data, no relevant differences could be detected in the period of 12 months regardless of the RV pacing burden (*peak exercise capacity:* lRVP: baseline: 105 ± 7 W, 6 months: 105 ± 6 W, 12 months: 114 ± 7 W, hRVP: baseline: 114 ± 12 W, 6 months: 118 ± 14 W, 12 months: 127 ± 15 W; *VO*_*2*_*max:* lRVP: baseline: 22.5 ± 0.8 ml/min/kg, 6 months: 22.8 ± 0.8 ml/min/kg, 12 months: 23.1 ± 0.9 ml/min/kg, hRVP: baseline: 21.4 ± 1.4 ml/min/kg, 6 months: 22.9 ± 1.9 ml/min/kg, 12 months: 24.4 ± 2.0 ml/min/kg; *VAT:* lRVP: baseline: 88.2 ± 6 W, 6 months: 85.8 ± 6 W, 12 months: 86.2 ± 6 W, hRVP: baseline: 82.0 ± 14 W, 6 months: 80.9 ± 14 W, 12 months: 85.0 ± 14 W).

## Discussion

The present pacemaker registry represents a detailed observational study regarding the influence of RV pacing on various echocardiographic and spiroergometric parameters in patients with preserved left ventricular function over a period of 12 and 24 months of pacemaker therapy. The main findings of this pacemaker registry were as follows: in the first retrospective part of the registry no relevant worsening of the LVEF and TAPSE could be observed within 24 months either in patients with a low RV pacing burden (< 40%) or in subjects with a high RV pacing burden with (> 40%). In the second prospective part of the current registry more detailed echocardiographic parameters including LVEDD, LVESD, LVEF, TAPSE, E/A-ratio and E/E′-ratio were measured 6 and 12 months after pacemaker implantation in order to reveal changes in the LV geometry and diastolic function in relation to RV pacing. Similarly, in the prospective study the comparison between subjects with low (> 40%) and high (< 40%) right ventricular pacing burden yielded no significant differences with regard to the echocardiographic parameters. These results were also reflected in the clinical short term outcome of the current registry, which was determined by spiroergometry. Accordingly, no relevant changes of peak exercise capacity, ventilatory anaerobic threshold and maximal oxygen consumption could be recorded independently of the RV pacing burden in the short term follow-up up to 12 months.

Controversial data exist concerning the long-term effect of chronic RVA pacing on the systolic LV function of patients with preserved LVEF. On the one hand the results of the current registry are in line with different previous studies. A large cohort study of pacemaker recipients with predominantly normal LVEF was also unable to demonstrate a clinically relevant decrease in LVEF due to RV pacing even after 44 months of follow-up [2]. Moreover, the randomized prospective PREVENT-HF Study did not demonstrate significant differences in several echocardiographic parameters after 12 months between RV apical and biventricular pacing for AV block [8]. In addition, a large registry of the Mayo Clinic could not reveal any statistically significant change in mean LVEF in long-term follow-up in patients with preserved LVEF who underwent AVJ ablation and RVA pacing [3]. On the other hand, in another large registry by Bansal et al. the incidence of pacemaker-induced cardiomyopathy, defined as decrease of ≥ 10% in LVEF caused by high burden of RV pacing was found to be 13.8% over a mean follow-up of 14.5 months [4]. In a further registry with 184 pacemaker patients as many as 22.8% of subjects developed a pacemaker-induced cardiomyopathy, with decrease in mean LVEF from 62.1 to 35.3% over a mean follow-up of 2.5 years [17]. Of note, in this study frequent RV pacing was defined as ≥ 20% RV pacing burden [17]. Thereby, the burden of right ventricular pacing and interventricular dyssynchrony were identified as the most important predictors for the development of pacemaker-induced cardiomyopathy [4]. Similarly to the current registry, in the previous randomized MOST and DAVID trials the RV pacing burden threshold for worsened clinical outcome was determined at 40%. Another study, however, also reported that pacemaker induced cardiomyopathy is not uncommon in patients receiving pacemaker for complete heart block with preserved LVEF and is strongly associated with RV pacing burden > 20% [18]. Nonetheless, there are no data to support any clearly defined limit below which RV pacing is safe and beyond which RV pacing is harmful [10].

It has been discussed that the location of the RV lead plays a role in the development of pacemaker induced cardiomyopathy. Some registries, nevertheless, have shown that non-apical RV pacing site does not offer any benefit in terms of incidence of pacemaker-induced cardiomyopathy over apical lead position [4]. Further, the Protect PACE study, which enrolled patients with complete heart block and preserved LVEF (> 50%) failed to demonstrate that RV high septal pacing was more beneficial than the standard RV apical pacing with respect to LVEF [19]. In addition, in patients with complete AV block with pacing-dependent rhythm, regardless of the pacing site, the paced QRS duration was a major determinant of the occurrence of pacemaker induced cardiomyopathy [20]. This finding is supported by the register of Khurshid et al. that showed that a paced QRS duration ≥ 150 ms was associated with the presence of pacemaker induced cardiomyopathy [17]. In the current registry all RV leads were implanted in a RV apical septal position. The QRS duration before and after pacemaker implantation was unfortunately not registered. Therefore, no conclusion can be drawn about the influence of the position of the RV lead and the paced QRS duration in the current work. Another factor in the development of the disease seems to be the total duration of pacemaker therapy. In the current registry no pacemaker induced cardiomyopathy could be diagnosed during a follow-up of 6, 12 and 24 months after pacemaker implantation. Previous registries reported an occurrence of pacemaker-mediated cardiomyopathy in the first 6–12 month of pacemaker treatment [1, 21]. However, this kind of cardiomyopathy could occur more than a decade after pacemaker implantation [22]. Therefore, the follow-up period in the current registry should have been adequate to register at least the early onsets of pacemaker-induced cardiomyopathy. Yet it must be critically noted that given a longer follow-up period more patients with a pacemaker induced cardiomyopathy could have been identified.

In addition to all the above-mentioned contributing factors, baseline LVEF appears to be one of the most important predictors for the development of a pacemaker-induced cardiomyopathy in various previous studies [3, 23]. This is absolutely consistent with the current study, which could not detect pacemaker-induced worsening of echo parameters in patients with preserved LVEF.

The LVEF also appears to play a critical role in the clinical outcome of RV pacing. Thus in the DAVID trial patients with standard indications for ICD therapy and an LVEF of 40% or less, RV pacing increased the combined end point of death or hospitalization for heart failure [7]. Furthermore, the Mode selection trial (MOST) could reveal an increased risk of heart failure and atrial fibrillation over a period of 6 years in patients with a cumulative RV pacing > 40% compared to patients with a low RV pacing burden [6]. Of note, in the MOST trial the clinical endpoints were not analysed with respect to the LV function. In addition, in the Multicenter Automatic Defibrillator Implantation Trial (MADIT) II study, RV pacing burden > 50% was associated with new onset or worsening of heart failure symptoms [24]. Due to the ongoing discussion about the adverse potential of RV stimulation in recent years, various pacemaker programming algorithms, switching from AAI(R) to DDD(R), have been developed to reduce unnecessary RV stimulation as much as possible [25]. However, it should be kept in mind, that the subsequent MVP trial (Managed Ventricular pacing vs. VVI 40 Pacing) failed to demonstrate non-inferiority of this algorithm [26]. Also a previous meta–analysis revealed that the reduction of unnecessary ventricular pacing fails to affect hard clinical outcomes such as permanent atrial fibrillation, all-cause hospitalization or all-cause mortality in patients with preserved left ventricular function [27]. In addition, it could be shown that the burden of RV pacing does not influence the quality of life in pacemaker patients [28].

Further, the incidence of atrial fibrillation as a sign of pacemaker-induced cardiomyopathy was significantly higher in patients with a high RV pacing burden compared to atrial-only pacing [29]. In the current registry the incidence of atrial fibrillation depending on RV pacing burden was not investigated. Nor were clinical endpoints such as the incidence of heart failure or atrial fibrillation chosen. However, changes in surrogate spiroergometric parameters for heart failure were explored. In accordance with the echo data no relevant changes of peak exercise capacity, ventilatory anaerobic threshold and maximal oxygen consumption could be revealed independently of the RV pacing burden in the short term follow-up of 12 months. Based on these data it could be proposed that in patients with preserved LVEF pacemaker therapy is unlikely to affect functional capacity. However, according to some literature it is not the RV pacing burden itself but the programmed AV interval which influences the exercise capacity [30]. Therefore, if a patient’s exercise capacity deteriorates under RV pacing, an echocardiographic optimisation of AV interval should be considered as primary non-invasive approach.

There are several therapeutic approaches to reduce the incidence of the pacemaker-mediated cardiomyopathy. In this context, the BLOCK-HF study suggests that patients with LVEF < 50% and AV block may benefit from biventricular pacing compared to RV pacing in terms of heart failure [31].

Similarly, in the long-term follow-up from the PACE study, HF hospitalizations were significantly increased among those receiving RV pacing compared to the CRT group [32]. Further, the 2-year results of the PACE trial showed that biventricular pacing minimizes LV dyssynchrony, preserves LV function, and reduces NT-proBNP in contrast to DDD(R) pacing in patients with high-grade AV block [9]. In contrast, in the echo-CRT study CRT therapy failed to prevent death from any cause or first hospitalization for worsening heart failure in patients with LVEF < 35% and QRS duration < 130 ms [33]. Another promising therapy concept for preventing or reversal of pacemaker-induced cardiomyopathy is the HIS bundle pacing (HBP) [34, 35].

Inspired by an excellent editorial written by Dr. Israel about his “top 10 excuses” with regard to unexpected results in prospective pacemaker studies, this study has several surmountable limitations as well [36]. First, the current study subjects were not randomised into the high and low RV pacing group. Second, the study population was too small to make valid conclusions about clinical endpoints like heart failure or mortality. Furthermore, the small sample size of the current registry negatively influences the power of the results regarding the echocardiographic and spiroergometric endpoints. Additionally, the current study cannot rule out long-term negative effects of RV stimulation, as the follow-up period did not exceed 24 months. Another limitation of this registry is the missing propensity matched analysis between the pacing groups to rule out potential sampling errors, which was not reasonable due to the small sample size. Another unusual aspect of the current registry is the divided design into a retrospective and prospective part. However, the expansion of the registry to a prospective part in order to include more detailed echo- and spiroergometric parameters is very useful for supporting the retrospective data.

## Conclusions

In summary, the current pacemaker registry regarding changes in echocardiographic and spiroergometric surrogate parameters of heart failure depending on RV pacing burden supports previous studies showing that pacemaker induced cardiomyopathy is a rare complication especially in patients with preserved LVEF in short term follow-up over 24 months. Despite this, screening for pacemaker induced cardiomyopathy should be performed particularly in the presence of new heart failure symptoms.

## Data Availability

The data that support the findings of this study are available from the first author, AY, upon reasonable request.

## Abbreviations

AFib: Atrial fibrillation
CAD: Coronary artery disease
HBP: HIS bundle pacing
hRVP: High RV pacing burden group
lRVP: Low RV pacing burden group
LV: Left ventricular
LVEDD: Left ventricular end diastolic diameter
LVEF: Left ventricular ejection fraction
LVESD: Left ventricular end systolic diameter
RV: Right ventricular
SSS: Sick sinus syndrome
TAPSE: Tricuspid annular plane systolic excursion
VAT: Ventilatory anaerobic threshold
VO2max: Maximal oxygen consumption
